# Engineering biology and automation–Replicability as a design principle

**DOI:** 10.1049/enb2.12035

**Published:** 2024-07-12

**Authors:** Matthieu Bultelle, Alexis Casas, Richard Kitney

**Affiliations:** ^1^ Department of Bioengineering Imperial College London London UK

**Keywords:** automation, bioinformatics, synthetic biology

## Abstract

Applications in engineering biology increasingly share the need to run operations on very large numbers of biological samples. This is a direct consequence of the application of good engineering practices, the limited predictive power of current computational models and the desire to investigate very large design spaces in order to solve the hard, important problems the discipline promises to solve. Automation has been proposed as a key component for running large numbers of operations on biological samples. This is because it is strongly associated with higher throughput, and with higher replicability (thanks to the reduction of human input). The authors focus on replicability and make the point that, far from being an additional burden for automation efforts, replicability should be considered central to the design of the automated pipelines processing biological samples at scale—as trialled in biofoundries. There cannot be successful automation without effective error control. Design principles for an IT infrastructure that supports replicability are presented. Finally, the authors conclude with some perspectives regarding the evolution of automation in engineering biology. In particular, they speculate that the integration of hardware and software will show rapid progress, and offer users a degree of control and abstraction of the robotic infrastructure on a level significantly greater than experienced today.

## Graphical abstract

Applications in engineering biology increasingly share the need to run operations on very large numbers of biological samples. Automation has been proposed as a key component for running large numbers of operations on biological samples. The authors make the point that, far from being an additional burden for automation efforts, replicability should be considered central to the design of the automated pipelines processing biological samples at scale—as trialled in biofoundries.

## 1 ENGINEERING BIOLOGY AND THE NEED FOR SCALE

### 1.1 Overview

Engineering biology—often called synthetic biology—is known for its reliance on core engineering principles, and its wide range of applications [1]. The field has been singled out as a key technology for the global transition to a bio‐based economy (bioeconomy) [2, 3]. In particular, it is expected to play a major role in the replacement of fossil‐based products and the greater use of renewable biological resources and wastes to produce chemicals, consumer and industrial goods, energy, and food [4].

Applications in engineering biology increasingly share a common practical need: that is, the need to run operations on biological samples at very large scales. This is the direct consequence of the application of good practices in engineering, the limited predictive power of existing computational models, and the desire to investigate very large design spaces in order to solve the hard, important problems the discipline promises to solve.

Automation, ‘*the use of* machines *and* computers *that can* operate *without* needing human control’ [5], has been proposed as a key component for running large numbers of operations on biological samples because of the feature most associated with automation: higher throughput [6]. Automation is a dual‐headed concept [7]. Mechanisation (mechanised automation) is the replacement of human muscle power (material and energy transformation), while computerisation (computerised automation) is the replacement of cognitive tasks (mental activity and sensory processes). Because of our interest in the processing of biological samples, in the paper we mainly focus on the mechanised side of automation—and only discuss software that supports mechanised tasks.

Higher throughput is not the only feature of automation of use in engineering biology. Because of the reduction of the influence of human operators, automation is also associated with higher replicability. Replicability is a core principle in engineering—and a *sine qua non* for any industrial translation effort. Lack of replicability will stop any such effort at its research and development stage, if not earlier. In this paper, we make the point that replicability should be made a core principle of the design of the automated pipelines processing biological samples at scale in engineering biology. We also show that this demand should not be seen as an additional burden for automation efforts, but as an essential requirement for the successful unlocking of the benefits of automation. In particular, there cannot be successful automation without effective error control.

### 1.2 Engineering biology

Several definitions of ‘synthetic biology’ have emerged since its inception early this century. One of the earliest, and most used, was offered by the UK's Royal Academy of Engineering: ‘*the design and engineering of biologically based parts, novel devices and systems as well as the redesign of existing, natural biological systems*’ [8]. A single definition has proved elusive however [9]. ‘Engineering biology’ is a recent term introduced by the UK Engineering Biology Leadership Council [10] and places a greater importance on translation and commercialisation: It is the ‘*overarching term that incorporates basic research and development (synthetic biology) and industrial translation*’, with a view ‘*to translate biodesign into commercially viable operations and to scale them out to deliver widespread economic prosperity and associated societal benefits*’. For simplicity, henceforth in the paper we will use ‘engineering biology’.

Engineering biology is known for its methodology, and its emphasis on the principles of engineering. Practically, the discipline rests on two pillars. The first pillar is a powerful set of abstractions and modelling components to represent biological systems, coupled with conventions and good practices for their use. These abstractions and components originate in the life sciences (molecular biology, biochemistry, genetic engineering etc.) and often are a consensus in these scientific disciplines. For instance, the central dogma of molecular biology introduces three types of biological objects: DNA, mRNA, protein. DNA and mRNA are represented by their sequence of nucleotides, proteins represented by their sequence of amino acids. Secondary and tertiary structures are also standardised. Early proponents of synthetic biology, inspired by design in electronics, advocated the use of a part‐device‐system hierarchy [11, 12] to describe the function of genetic elements. The adoption and expansion of this early form of functional analysis has since given rise to modern biodesign. The existence of these abstractions is a testament to the level of understanding of biological systems accumulated throughout the years, and to the power of the reductionist approach in biology. But it is easy to underestimate the power this offers—nothing less than the ability to undertake systematic design, and a modelling language for biological processes. And it is upon this language that the second pillar is built: the ever‐growing set of foundational technologies, conventions, good practices and standards, used to build and design the ‘*biologically based parts, novel devices and systems*’.

The demonstration that biology is amenable to systematic modelling, the development of powerful foundational technologies, and a distinctive methodology, make the discipline stand out from other areas of biotechnology—and provide strong backing for claims of a genuine paradigm shift [13].

### 1.3 The need for scale in engineering biology

**FIGURE 1:**
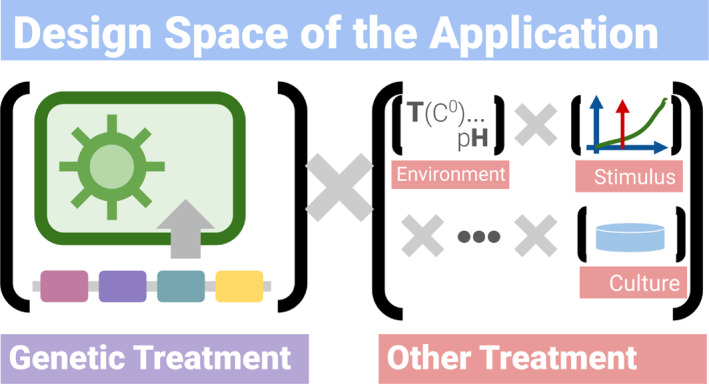
Design space in engineering biology. The design space is the set of all combinations of the independent variables. It can be split into a subset with all the variations in the genetic design, and another including experimental conditions and protocols (parameters and sequence of actions).

Applications in engineering biology often share common practical needs. For example, the need to build a large number of biological designs sharing a common genetic design; and the need to run experiments/assay at large scales as part of their development. These needs are the direct consequence of three factors:

- **The need to investigate large design spaces** (Figure 1). Biological applications require the controlled variation of the genetic content of a system, in conjunction with the variations of other study/design factors—either to optimise the properties of the system or to ensure some measure of stability with regards to practical conditions.
- **The need to tackle biological complexity.** This is due to the intrinsic complexity of biology, the insufficient predictiveness of models, and lack of principles for biodesign.
- **The application of good practices regarding design, testing and validation.**

The need to investigate large genetic design spaces can be illustrated with a simple example from metabolic engineering—to be more precise from its most engineering‐influenced flavour of combinatorial pathway engineering [14, 15]. The approach uses libraries of functional, preferably characterised [16, 17], components to introduce genetic diversity [18, 19, 20, 21]. It is very appealing because it yields, with only a few degrees of freedom, a large number of levels over which to tune intricate pathway expression. Because it enables a systematic, multifactorial approach, it does not require a deep knowledge of the pathway as the classic debugging approaches [22], which seek to remove pathway bottlenecks. But large design spaces quickly become inevitable with such a strategy. With a 5‐gene pathway such as violacein [23, 24], a simple RBS‐based optimisation yields 100,000 possible combinations for a small library of 10 RBS. When designing biological components, the number of possibilities becomes enormous. A simple promoter with 50 base pairs yields 4^50^ possible sequences, that is, a little bit over 10^30^ combinations—most of them ineffective as promoters.

In addition, there is a need to test the constructed biological systems for a combination of variations, including experimental conditions and protocols (parameters, and sequence of actions). Such testing may be carried out for the purpose of optimisation. For instance, the production of a metabolite of interest can be maximised by altering the type, timing and magnitude of an external stimulus. Alternatively, testing may be carried out to assess the stability of a design for a set of conditions such as a range of temperatures. In some cases, for example, the optimal combination of the components of a cell‐free system [25], this can represent a substantial augmentation of the design space. In the rest of the paper, we will use the term ‘treatment’, which is widely used in statistics, to mean the set of all the independent variables in the design of the application, and in whose effects the designers are interested. In engineering biology, one of its subsets consists of all the variations in the genetic design–we will call it the ‘genetic treatment’.

The second point, tackling biological complexity, is reflected in the history of biodesign. Early this century, engineers advocated that engineering biology mirror the design abstraction hierarchy used in electronic circuit design. The appeal of this approach lay in its simplicity and its proven success with early toy systems [26, 27]. This spurred a range of characterisation efforts [28, 29] and the creation of repositories such as the iGEM Registry of Standardised Biological Parts [30], Addgene [31], SynBIS [17] and SynBioHub [32]. But it has now been shown that the assumptions behind modular and hierarchical design were often not met, and the performance of parts was highly context‐dependent and lacked predictability [33, 34, 35]. The breadth and impact of contextual issues is noteworthy: different behaviours in different growth media, pH or temperatures [36, 37], DNA supercoiling [38], resource sharing [39], growth rate feedback [40, 41], positional effects of the components [35], integration method [42, 43] etc.

Biodesign still lacks a user‐friendly framework for design and troubleshooting [34]. Ilya and Del Vechio's suggested some guidelines [44]starting from a point assumed free of contextual effects, and then, iteration after iteration, model the interactions and compensate for them. Without validated models with good predictive power, even this approach to biodesign requires a great deal of assays to test and validate the designs.

Finally, unwanted variations need to be accounted for. Engineering biology is different from other fields of engineering—in that biology is intrinsically stochastic [45, 46]. For any given treatment, the behaviour of the system must be estimated by aggregating the measurements of several repeats, ideally to achieve some statistical significance. This simple requirement is often overlooked, as it is often unwieldy. A common heuristic with statistical tests such as *t*‐tests recommends using at least 30 samples; a handful of repeats are used instead. Good practice also suggests using design of experiment (DoE) methods to account for confounding factors. Methods such as blocking need additional samples; while other methods (e.g. randomisation) complicate the way the assays are run.

Engineering biology has to contend with some very specific demands of biological materials. Despite popular analogies [12] likening biochemical processes to computations (cells to computers and tissues to networks), biological samples and processes are subject to very specific constraints:

- **Biological processes are stochastic and irreversible.** They cannot be stopped and resumed. Changing their speed may change their outcome.
- **Biological samples cannot be duplicated like data.** Nor can they be built de novo to tight margins of error as for electronic components. Also, biological samples cannot be broken into subunits to be processed in parallel and then recombined.
- **Biological samples mutate with time.** They divide and replicate imperfectly. Their genetic content is liable to mutate and they are subject to ageing.

The consequences for the processing of biological samples are important. The processing of biological samples is irreversible, and subject to biological noise. It cannot be repeated. Failure is terminal. Different conditions cannot be applied to the same sample. The only form of repetition available is to create approximate copies of the samples through the error‐prone replication mechanism of the cells. Also, processing cannot be parallelised. The outcome is liable to change if any condition changes. Finally, the outcome will depend on the time when processing takes place and the protocol used.

It is against this backdrop of a rising need to process biological samples at scale, under specific constraints, that we discuss the use of automation in engineering biology. Specifically, we discuss how automation helps with regard to high throughput and replicability.

## 2 LABORATORY AUTOMATION AND HIGH THROUGHPUT

### 2.1 Laboratory automation and high throughput

The first aspect of automation we wish to discuss in the context of engineering biology is the feature most associated with automation in general: higher throughput [6].

To do this, we will first discuss the history of automation in the life sciences and biotechnology. The pharmaceuticals industry has been a key player in the development and adoption of laboratory robotic equipment due to their need for testing compounds against a very large number of targets—so much so that the history of laboratory automation parallels the development of modern drug discovery [47]. This need was identified as early as in the mid 1980s when large Streptomycete libraries were successfully constructed and needed testing for antibiotic production under a variety of fermentation conditions [48]. In industrial terms, the stakes were high. Those pharmaceutical companies that had the ability to test quickly and efficiently had a genuine competitive edge over the pharmaceutical companies that could not. However, in the early 1990s, testing a collection of 250 k compounds would still take 2 years [49] at the state‐of‐the‐art rate of 2500 a week.

It is the gap between the pharmaceutical industry's needs for rapid drug discovery and screening, and the capacity of even the most advanced companies, that led to the birth of the discipline now known as high‐throughput screening (HTS) [47]. Technologies already used in medical diagnostics (robotics, liquid‐handling devices, automated measurement devices, system control etc.) were adapted to achieve the necessary integration of compound supply, assay operation and data management [49].

The standardisation of microplates [50] was a key catalyst in the development and adoption of automated platforms [49]. In the early 1990s, 96‐well microplates became the main compound‐handling and screening format in the industry. Assays were subsequently adapted to higher‐density microplates (384‐well, 1536‐well and higher), resulting in a significantly higher ability to process samples in parallel [51]. This in turn has led to a significant reduction in the quantities needed for the assays: while assays in single tubes need micrograms of compounds, assays on 1536‐well plates only use a few micrograms [49]. It is noteworthy that after automation and miniaturisation were leveraged to increase capacity, HTS focused on factors such as process quality, time, and cost [52], so more targets could reliably be identified. This was followed by a third phase with more flexible, target‐specific, screening strategies [53]. These trade‐offs between throughput, cost, time and data quality, and the need for smart techniques to navigate the large spaces will be familiar to anyone in engineering biology.

The wide adoption of robotic equipment in the pharmaceutical industry has been a clear source of inspiration for engineering biology. However, despite early claims that automation was revolutionising biology, or was going to [54], the promise remains unfulfilled. Academic laboratories have only minimally embraced automation tools [55]. The poor state of adoption should be seen against a seemingly favourable background. Many techniques employed in engineering biology are routine [55] and have been implemented in robotic labs [6]. Reasons for the slow adoption of automation include:

- **Costs.** Setting up and running an automated infrastructure comes with high costs. Expenses include purchasing the automated robotic and high throughput equipment, all the consumables, and the software and support for the staff [56].
- **Expertise and training limitations.** Automated infrastructures require new ways to work, which, in turn, requires additional training for the staff [55]. Expertise limitation was identified as an obstacle in HTS. The decision then was to create islands of automation [49].
- **Additional time for set‐up and optimisation of new protocols** [55]. Automation should therefore be saved for tasks worth automating—for instance tasks that will be performed a large number of times.
- **Lower precision of robotic platforms** [55, 57].
- **Lack of flexibility.** Flexibility, defined as ‘*the capability of an automation system to be adapted to a new protocol with the minimum cost and downtime of the existing system*’ [58], is a critical requirement for research laboratories–where protocols are continuously modified. Commercial products are notoriously difficult to reconfigure. This has been one of the most frequent complaints of laboratory automation system users [58, 59]. New generations of hardware manufacturers such as Opentrons [60] now put flexibility and programmatic access first [61].

Despite—or maybe because of—the low level of automation in engineering biology, efforts proceed at a fast pace to unlock the benefits of automation. High‐throughput platforms are in greater use [62], and advanced development workflows, such as the design‐build‐test‐learn (DBTL) workflows, have been successfully automated [63, 64]. It is worth stressing that these advances have taken place on the more open platforms—indicating that some of the obstacles for a wider use of automation are being lifted. More studies are being published that use automation to achieve higher throughput. For example, Goldman and co‐authors [65] used automation as part of a DARPA‐backed replication study, which, in their opinion, allowed them to scale up the experiments ‘*by two orders of magnitude*’. The construction and cloning phases have been successfully automated, yielding significant improvements. The Edinburgh Foundry [66] has reported 20× improvements for DNA assembly reactions [67]. Another biofoundry, iBioFAB, has reported 10× improvement with their automated, multiplex genome‐scale engineering of *Saccharomyces Cerevisiae*. With automation, they could build up to 1000 TALEN constructs per day at <$3 each—a 300× reduction in cost [68].

### 2.2 Biofoundries as drivers of laboratory automation

Among the initiatives to spread the benefits of automation, the most comprehensive was the establishment of a network of biofoundries called the Global Biofoundry Alliance [67]. Some private companies also run their own biofoundries—for example, Lesaffre [69] and Ginkgo Bioworks [70]. Although they are more limited in scope (R&D and commercialisation), most of the points in the discussion here also apply to these private sector biofoundries. Biofoundries are specialised laboratories that combine software‐based design and automated or semi‐automated pipelines to build and test genetic devices [71]. To enable the rapid design, construction and testing of genetically modified organisms, biofoundries are organised around the DBTL cycle [67].

Generally, their purpose is to help accelerate commercialisation of synthetic biology products and biomanufacturing process engineering and the transition towards sustainable bioeconomies and development bioeconomy [72]. This, in turn, requires the creation of ‘*beyond‐the‐lab*’ capabilities tasked with application development, scale‐up, and commercialisation [73, 74, 75]. Biofoundries occupy a very specific place in the innovation ecosystem of their countries. Farzaneh [76] likened them to *nucleating hubs*’. They generate and support webs linking academic researchers, industry (one of their core missions is to provide access to startups and SMEs to expensive, specialised equipment) and policy‐makers (government, standard bodies, and funding agencies). An example of such a hub is the London Biofoundry at SynbiCITE [77]—the UK's industrial translation centre for synthetic biology/engineering biology. The need for such hubs has now been widely acknowledged by policy makers. Over 50 countries, including all the G7 nations, have adopted national strategies related to the bioeconomy and biofoundries [56, 75].

Biofoundries are the natural continuation/evolution of all the efforts of automating mechanised laboratory processes. They offer physical locations, where all the constituent processes can be physically concentrated and integrated. Biofoundries typically contain equipment ranging from liquid‐handling equipment, DNA sequencing, automated controllable bioreactors to high‐throughput analytical equipment—and are operated by specialised staff. They are also ideal testbeds for the integration of automated robotic platforms and dedicated software tools. These tools go beyond laboratory information management systems and support all stages of the DBTL cycle. Three classes of DBTL tools are of particular note:

- **Biological design tools.** In conjunction with repositories of genetic parts, they let users generate large design spaces [78, 79, 80], and support their construction. These two in practice support the first two phases of the DBTL cycle.
- **Computational tools for the learning phase of the DBTL cycle.** They suggest new experiments to investigate large design spaces efficiently. Many implement DoE strategies or optimal experimental design [81, 82]. Machine learning tools also fall under this category [83].
- **Equipment programming software and interface drivers.** These tools remain very piecemeal. The SILA2 standard (standing for ‘Standardisation in Lab Automation 2’) [84, 85] has been adopted by some vendors to enable interoperability between pieces of equipment [86]. SynBioPython [87] is an initiative launched by the Global Biofoundry Alliance for all their software projects. Other solutions have been put forward for laboratory workflow automation, either commercial like Antha [88] or open source, such as Aquarium [89].

**FIGURE 2:**
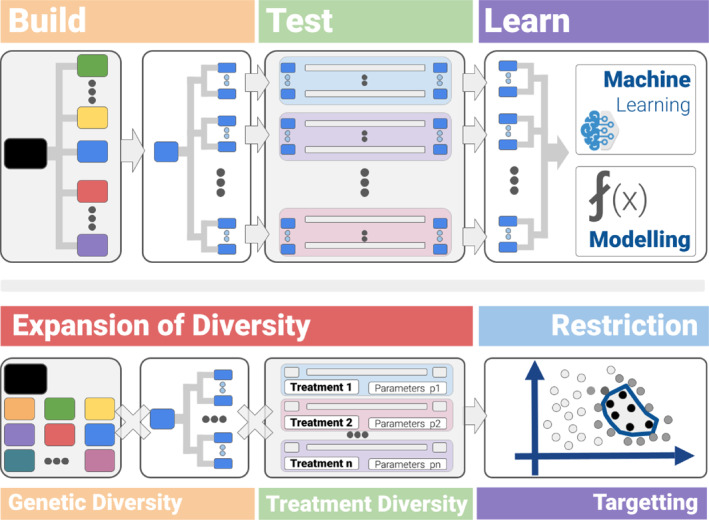
Implementation of the DBTL cycle in biofoundries. The DBTL cycle uses an expansion‐reduction strategy. The expansion portion (build and test phases) enables the controlled introduction of variations into the system, and does so in decoupled stages, to yield a combinatorial effect. The reduction portion (learn phase) identifies its most promising regions, so they can be targeted in later rounds.

The structure of the DBTL cycle shows why it is so powerful. The cycle uses an expansion‐reduction strategy (Figure 2). The expansion portion (build and test phases) enables the controlled introduction of variations into the system, and does so in decoupled stages, to yield a combinatorial effect:

- **Build phase.** The phase encompasses the genetic diversity necessary for the development of the application of interest. In practical terms it consists of the construction of libraries of genetic variants—often assembled by reusing previously generated libraries. For instance, a library of plasmids can be assembled from existing libraries of components; or a bacterium can be transformed with previously assembled plasmids.
- **Test phase.** This phase comprises two sub‐phases: a sample preparation phase, and an assay phase. The sample‐preparation phase generates the samples from biological material that was previously generated in the build phase. Variations in treatment (environmental parameters, stimuli type and magnitude etc.) are introduced in the assay phase. Samples are grouped and processed according to their genetic content and the treatment they receive.

The reduction portion (learn phase) reduces the need for brute force investigation of the design space and identifies its most promising regions, so they can be targeted in later rounds.

The power of the biofoundry approach lies in the effective targeting of the steps of the expansion portion of the DBTL cycle for mechanised automation. Biofoundries treat these steps as an opportunity for throughput multiplication and have automated the necessary workflows. The engineering principles of standardisation and reusability play a key role in the approach. Common DNA assembly workflows, already automated in biofoundries [90, 91, 92, 93], use reusable biological components. The overall construction can be split into several stages performed by separate teams. Automated operations also use standardised protocols. The result is an effective trade‐off between the biological constraints, the resources of the biofoundry and the demands of the study.

## 3 AUTOMATION AND REPLICABILITY

### 3.1 Replicability

The other aspect of automation we wish to discuss in the context of engineering biology is replicability. Reproducibility, replicability, repeatability (the terms are often used, somewhat loosely, interchangeably) are key principles of the scientific method. For the findings of a study to be recognised and be considered by the wider research community, they should be repeated, preferably by separate researchers, with a high degree of reliability [94]. Replication studies vary, depending on what methodological factors they share. Some replication studies use identical methodologies and, ideally, identical equipment and protocols—they can then be considered straightforward copies of the original study. Conversely, some replication studies use different methodologies. Replication using both identical and different methodologies is very strong evidence for the validity of the result.

Concerns about lack of reproducibility and replicability have been recently expressed in both scientific [95, 96] and popular media [97]. A landmark editorial in Science [98] bluntly stated ‘*Recently, the scientific community was shaken by reports that a troubling proportion of peer‐reviewed preclinical studies are not reproducible*’. A subsequent Nature survey [96] of 1576 research scientists found that almost 70% of researchers (78% among biologists) had attempted, but failed, to reproduce other researchers' results in the laboratory. Worse still, 60% failed to replicate their own research. This state of concern is now referred to as the ‘replication crisis’ and has led the National Academy of Sciences (NAS) to establish a committee on the ‘*Reproducibility and Replicability in Science*’ [94]. In this paper, we use the definitions of reproducibility and replicability contained in the NAS report. Although definitions of reproducibility and replicability vary across science and engineering [99], the NAS definitions (See Table 1) are extremely powerful—as they decouple the concepts and make them complementary. Reproducibility is a quality feature of the analysis of the dataset (is the conclusion valid given the collected data?). Replicability is a quality of the outcome of the study (is its conclusion valid? Can it be reliably used in another setting?). Failure to reproduce results indicates issues with the study, including corrupted data or bugs in scripts. Failure to replicate results can result from sources ranging from faulty design (or worse, fraud) to natural randomness (possibly exacerbated by a lack of control of some variables) to, excitingly, a new discovery.

**TABLE 1:** Repeatability, reproducibility and replicability as defined by National Academy of Science (NAS) and the Association for Computing Machinery (ACM).

Term	ACM definition	NAS definition
Repeatability	*‘The measurement can be obtained with stated precision by the same team using the same measurement procedure, the same measuring system, under the same operating conditions, in the same location on multiple trials.* *For computational experiments, this means that a researcher can reliably repeat her own computation.*’	
Reproducibility	*‘The measurement can be obtained with stated precision by a different team using the same measurement procedure, the same measuring system, under the same operating conditions, in the same or a different location on multiple trials.* *For computational experiments, this means that an independent group can obtain the same result using the author's own artefacts.’*	*‘Reproducibility is obtaining consistent results using the same input data; computational steps, methods, and code; and conditions of analysis.* *The definition is synonymous with “computational reproducibility”’*
Replicability	*‘The measurement can be obtained with stated precision by a different team, a different measuring system, in a different location on multiple trials.* *For computational experiments, this means that an independent group can obtain the same result using artefacts which they develop completely independently.’*	*‘Replicability is obtaining consistent results across studies aimed at answering the same scientific question, each of which has obtained its own data.* *Two studies may be considered to have replicated if they obtain consistent results given the level of uncertainty inherent in the system under study.’*

*Note*: The definitions from the ACM [100] have been included for comparison.

Despite its critical importance, there has been limited investigation into the replicability of publications in life sciences [55]. This also holds in engineering biology [101], where the most prominent replication studies have been in the context of iGEM interlab studies [102, 103, 104], and DARPA [65]—using identical methodologies, but with noticeably different outcomes. A review on the topic of the estimation of the relation between mRNA and protein abundance [105] identified a wide range of causes for the discrepancies between published results. These causes predictably included methodological differences such as calibration issues. Noticeably, the discussion also included biological considerations such as temporal and spatial scales (e.g. cell populations and tissues vs. single cells), which had such an effect in practice that some studies did not measure the same properties.

Replicability is a core principle in engineering [55], and crucial for any industrial translation and production effort. The rapid development and manufacturing of COVID‐19 vaccines on a global scale [106, 107] would never have been possible without a concurrent emphasis on replicability during the development and testing phases. This was equally true during the manufacturing phase that involved hundreds of sites manufacturing the vaccines and their components. Failure to replicate can be terminal to R&D efforts—more generally, insufficient replicability hampers such efforts. High‐throughput screening once again offers an example [108], as it has been accused of being a contributory factor to the decline in reduced productivity in the pharmaceutical industry (poor‐quality data and insufficient hit rate).

The link between automation and higher replicability can be described in an idealised scenario as follows. First, automation leads to higher throughput. As previously discussed, this leads to more processed samples, more collected data and a better statistical analysis. Second, automation reduces the input of human operators. Since human activities are significantly affected by stress, tediousness, repetition and fatigue [55, 109], mechanisation is expected to lead to lower error rates for these operations—especially for higher throughputs. Mechanisation is also expected to yield more homogeneous outputs. Humans vary considerably in their ability to learn and perform new motor skills [110] indeed, while some skills, such as micropipetting, have a massive influence on the output of experiments [111]. Note that mechanisation should not necessarily lead to more precise outcomes; occasionally, a skilled technician will perform more accurately.

The advantages of a reduction in human factors are not solely limited to mechanised automation. Some actions may be performed by a trained operator without software support, but the cognitive load and labour involved makes doing so inefficient even at modest scales. For instance, the generation and management of all construct sequences is best left to a dedicated infrastructure [112]. Quality control for construction steps are also best automated with a dedicated sequence‐matching software [113]. Also, while some tasks are not automatable, others can only be performed by machines/computers [114]. The design and learn phases supply many examples of such tasks: for example, bioinformatics tasks such as a BLAST database search [115], or the construction of a phylogenetic tree [116].

### 3.2 Replicability in practice–A case study

The relationship between automation, high throughput and replicability is, unfortunately, more complicated than the basic version in Section 3.1. Poor replicability can easily spoil automation efforts. As appealing as reducing human influence and increasing throughput are, careless deployment of automation is liable to introduce new uncontrolled errors and new confounding variables. The results can be contaminated, or even result in useless data. These pitfalls of automation can, for example, be illustrated with a recent study conducted by the authors of this paper [117] on the topic of the automation of liquid handling.

Liquid handling is the most common step in all protocols in engineering biology. Liquid‐handling operations are labour‐intensive and inefficient when performed manually [58]. Liquid‐handling platforms are therefore central to the transformation of research laboratories and their adoption of automation. Robotic liquid‐handling platforms all have the usual automation advantages over manual operations: they increase the throughput and perform consistently and without fatigue [118]. Achieving reliable, robust, repetitive, and high‐speed operation is not without its challenges. A wide range of physical factors play a role in the dispensation of liquids with air‐cushion pipettes. Ewald [119] listed density and viscosity of the pipetted liquid, pre‐wetting and environmental factors such as relative humidity, vapour pressure, system temperature, air pressure and altitude as the most important. To compensate for the influence of these factors, calibration is often necessary [120]. Some factors such as evaporation control, viscous material handling, calibration, clogging detection, air bubbles dissolution and system integration [118] are harder to compensate for. Clogging of tubes, valves and pipetting heads can be caused by viscous material, dust and particles or accumulation of liquid debris. Air bubbles may form during aspiration or dispensing and cause inaccuracies in volume handling.

Although valid low‐level explanations, these factors are not practical enough to help with protocol design and testing. Albert [121] took a more actionable view of liquid‐handling errors and developed an error classification framework, linking physical and mechanical factors with observable effects. The framework is based around three sources of errors:

- **Equipment related errors.** The types of tips are critical to the accuracy of volume transfer—material, shape, fit, and wettability being the most important factors. A tip unsuited to the material being dispensed can also cause random contamination.
- **Pipetting Methods.** Forward mode, where the entire aspirated reagent in the tip is discharged, is suitable for aqueous reagents with or without small amounts of proteins or surfactants. Reverse mode, where more reagent is aspirated into the tip than is dispensed, is suitable for viscous or foaming liquids. Pipetting methods are generally unsuitable to liquids that are non‐aqueous or complex (i.e. with one or more components or additives, such as salts, sugars, detergents, surfactants, or proteins).
- **Liquid‐dispensing schemes.** The sequential dispensing scheme first aspirates a relatively large volume of the reagent, before dispensing the reagent into several wells as per protocol. Tips must not touch any liquid present in the wells to avoid contamination or dilution. The serial dilution scheme only travels back and forth between wells and the reservoirs. Issues occur if the reagents are not properly mixed before the transfer, and also include potential contamination. Iterative schemes transferring between wells on the same plate (for instance, column by column) spread errors across the plate and are contamination‐prone.

Albert's framework led the authors of this review to conduct their own study in the London Biofoundry [122] at SynbiCITE, and to investigate the reliable automation of liquid handling and identify the classes of errors introduced. They did so from the standpoint of the construction of the standard curve for lycopene in dimethyl sulfoxide (DMSO). Since lycopene is a carotenoid of industrial interest and DMSO is a popular extractant, the study also had industrial relevance. The development of a standard curve necessitated the development of flexible liquid‐handling methods that were generalisable to other automated applications. Because lycopene in DMSO is a complex, atypical mix, it magnified and revealed issues with automated liquid‐handling protocols—and stress‐tested them.

Once the protocols were confirmed, they achieved higher throughput and could use data analysis methods, which was necessary due to the challenging nature of the mix. Probably the most generalisable aspect of the study related to the control of errors. Several sources of error were identified during development of the protocol, which had to be separated by design, and tightly controlled with their own dedicated protocols:

- **Mix preparation.** The age of the mix was a major confounding variable.
- **Dilution errors.** Some dilution schemes were proven superior to others. A popular iterative dilution scheme was shown to introduce new correlated errors, and was, at best, only to be used for scoping. Direct dilution was shown to be very suitable, due to its predictable errors and its highly programmatic nature.
- **Measurements.** The way measurements were conducted had to be adapted to the properties of the mix. In particular the time scale of the measurements and the number repeats had to account for all solubility issues.

The study also identified several additional topics worth noting. First, even for such a simple application, plate design quickly became beyond manual implementation, due to the associated human cognitive load. Second, the importance of some factors of the study on the outcome of the assays was such that, in addition to precise protocols for their control, the authors recommended monitoring the value of these parameters. This effectively introduced additional quality control steps. Finally, there was a clear need for a communication strategy, so anyone willing to reuse the constructed curve, or recreate one on their own setup, could do so reliably and not introduce uncontrolled errors into their own pipeline.

Even at its modest scale, the study showed that replicability and error control should be taken into account from the beginning—when an automated pipeline is developed and deployed into production. This is important, because it implies that successful automation requires more than solving the reasons for limited adoption of automated platforms (listed in Section 2.1), and which are being resolved already, as equipment is getting more flexible and more precise, and expertise limitations ease as dedicated training programs are developed.

## 4 REPLICABILITY‐SUPPORTING INFRASTRUCTURES

In this section, we discuss how a dedicated IT infrastructure should be used to support automation, and why replicability should be a core element of its design.

For the sake of simplicity, we assume that an automated pipeline has already been designed and validated. And, we will focus on using it to produce biological samples, run assays on the samples and produce data at large throughput. Many of the ideas and principles we will describe also apply to the development of automated protocols, and the transfer of protocols to another setup or facility. Elements of the infrastructure can still be used to prevent the introduction of higher‐level errors into the development process. Effectively, protocol development can be modelled as a design study (as shown in Figure 1). But instead of a design space comprising many genetic variations, the design space then mainly consists of protocol parameters.

By ‘*designed and validated already*’, it is assumed that pipeline achieves the desired goal in terms of throughput and quality of outcome. Variations of outcomes are then a combination of the stochasticity of the biological systems and process variations (e.g. measurement accuracy), and are considered acceptable for the task at hand. Process variations will decrease as robotic platforms improve; biological stochasticity on the other hand will not.

The pipeline may be only partly automated and, therefore, subject to significant influence from human operators. It may be organised around islands of automation. Or it may be fully automated, and free from human influence. But its design and good operation rely on a core assumption: all the protocols governing the operation of the pipeline are strictly adhered to.

We call unwanted deviations from this assumption higher‐level errors, to distinguish them from the expected low‐level process errors—which the design of the pipeline has striven to control. Examples of higher‐level errors abound in the industrial world. Food poisoning may occur in fast food restaurants caused by a break of the cold chain outside of the premises. In the medical world, dosage and prescription errors can be devastating and are tragically common [123]. Such errors can appear in even the most sophisticated environments. Misconfigured systems bring down popular websites. In 1999, after almost 10 months of travel to Mars, the Mars Climate Orbiter burnt and broke into pieces—because someone failed to use metric units! Even activities based on mastering probabilities of events can fall foul. Casinos, so adept at controlling risk when it comes to their customers, fall victim to less predictable events such as staff misbehaviour—endangering their gambling licences in the process. In engineering biology, higher‐level errors include:

- **Direct human input errors.** These errors may be punctual and considered independent. Or they can be the product of repeated operations under fatigue, and therefore liable to be correlated. Responsibility may be shared, as in the case when some settings were changed by a previous user, but the operator failed to check.
- **Instrument failures.** These errors may affect a few wells or entire plates, and range in severity from partial (the hardest to detect) to complete (the easiest to detect).
- **Unexpected data.** Instruments may return unexpected values, or no values at all. As with the instrument failures, these errors may apply to a few wells or entire plates.

More subtle errors relating to drifting conditions in the pipeline and failure to conduct checks for the drifting conditions may occur. This is typically the case with the calibration of instruments. In the previously‐mentioned liquid‐handling study, the researchers were forced to add quality control steps into their pipeline to make sure of the state of the mix of lycopene and DMSO. More generally, protocols should be applied in their entirety. Although this is beyond the scope of this discussion, there is a clear need to develop effective communication strategies in engineering biology to impress on all the participants the importance of the protocol steps and the influence on some of their parameters on the outcome.

In all cases, these higher‐level errors are caused either by human operations, unvalidated software operations, or hardware malfunction. The cause for these errors may be very far removed, if they took place much earlier in the workflow or in other locations. They result in irrecoverable operations on samples—not to mention wasted resources and time. If the errors are not spotted in time, which is often hard to do, this will result in bad data being collected and poorly replicable results.

It is the authors of this paper's opinion that any dedicated IT infrastructure aiming to support automation—beside the usual mission of storing experimental and procedural data, and letting scientists visualise them and compute what analytics is deemed necessary—should also be actively involved in the reduction of high‐level errors and nuisance human factors so fewer assays are wasted, and collected data are of higher quality.

Such an infrastructure would go beyond that presented by Peccoud and co‐authors [124], which is only concerned with the logging, storage and subsequent statistical analysis of all experimental and procedural factors ‘*to unwind how different factors contribute to the variation of data by planning and documenting data collection which help associate the controlled variable of interest with the observed variation in experimental outcomes*’. The capacity to run *post hoc* analysis, which Peccoud and co‐authors demonstrate with identification the influence of media preparation on cell viability in their process, is obviously a requisite for an infrastructure supporting replicability. But it does not help with ensuring/enforcing that protocols are followed and that the assays are conducted as intended. At best, it can only identify when the protocols were not followed—provided the relevant information was logged.

**FIGURE 3:**
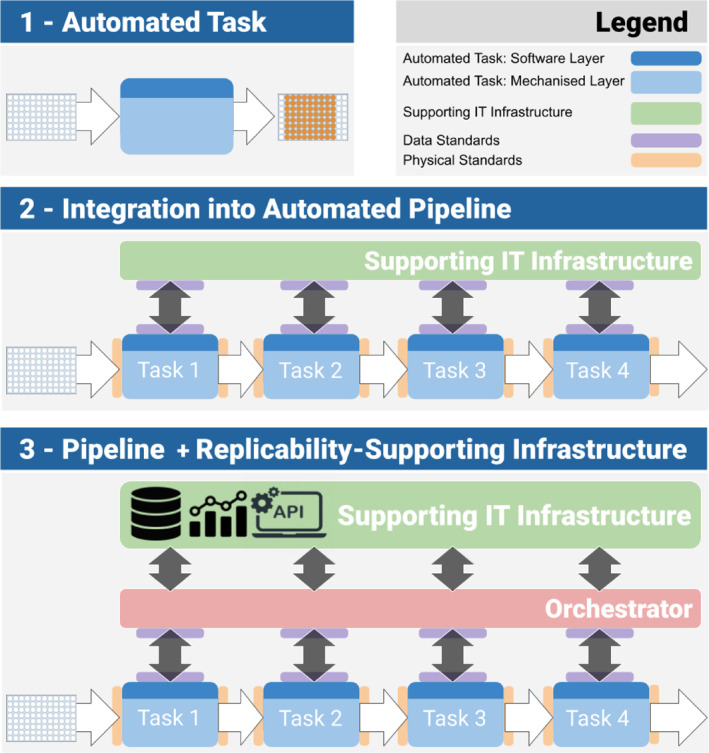
Automated tasks and automated pipeline. (1–Top): Every automated task has two components—a software layer, coupled to a mechanised layer. Instructions for the task are communicated to the mechanised layer by the software layer. (2–Middle): Multiple tasks are connected into an integrated automated pipeline. Information flows are denoted by black arrows, and flows of physical objects by white arrows. There is now a chaperoning IT infrastructure in charge of collecting all the generated data and storing them. (3–Bottom): the IT infrastructure needed to support replicability contains an orchestrator, charged with sending the instructions to the various automated platforms, monitoring the advancement of the workflow, and validating the content of the information passed between components.

Figure 3 presents a high‐level model of how automation of workflows currently takes place in a biofoundry (the most likely setting for the deployment of such an infrastructure), and how the software infrastructure, and its components, should evolve to fully support automation. Automation of a workflow involves the integration of separate automated tasks into automated pipelines. Every automated task has two components—a software layer, coupled to a mechanised layer as shown in Figure 3 (top section). Instructions for the task are communicated to the mechanised layer by the software layer, so that the automated platform performs the specified task. Practically, this corresponds to the automation of a single task—at best an island of automation. We call this ‘Level 1 integration’. Figure 3 (middle section) shows a generic workflow, with four tasks performed sequentially on one microplate, and integrated into a single automated workflow. A typical example would be plate creation; culture followed by the application of a stimulus; another round of culture; and finally some measurement. Information flows are denoted by black arrows. Flows of physical objects (the microplates) are denoted by white arrows. As previously, the instructions for any task in the workflow are transmitted to the software layer of the task and then to its mechanised layer. There is now a chaperoning IT infrastructure that oversees the collection and storage of all the data (operational and experimental data). This is the type of infrastructure described by Peccoud and co‐authors, which we call ‘Level 2 integration’.

Finally, Figure 3 (bottom section) presents the type of IT infrastructure, which we call ‘Level 3 integration’, needed to support replicability. Note the addition of the orchestrator. Beside collecting all the generated data and storing them, the orchestrator also sends the instructions in the order of the workflow; monitors the advancement of the workflow; and validates the information passed between components. The infrastructure is built on three sets of technologies sharing two common attributes:

- **Efficient interfacing.** They interface the modular components of the experimental workflow, the components of the data flow, and the instructions of the user(s).
- **Standardisation.** They standardise these interfaces, and offer opportunities for validation against initial specifications, and thus error‐control.

The first set of technologies concern data management and information exchange. Data will need to be structured in a way that reflects the history of transformations (lineage) of the plates and their samples—and makes this history available for data analysis and monitoring. The infrastructure should also deal with operations related to data integrity and standardisation. These are backend‐type operations that may not seem of major importance in a scientific study, but carry a significant cognitive load and cause practical errors when conducted at even modest scales. These include data import (from local or external sources, or from the various measuring devices), identifying missing or erroneous data (e.g. with the wrong data type), cleaning them, before converting them to a settled format and storing them. The use of standards is an essential aspect of improving replicability. Standards are the enablers of all integration efforts. They act like invisible glue between the different processes in the experimental pipeline. Several data standards are in wide use in engineering biology—together with more general standards in communication systems and computing. These standards are shown in purple in Figure 3. Supporting common, existing data formats is particularly important when sharing data and results—hence, indirectly, for replicability. Data standards of note in engineering biology include sequence standards, such as SBOL [125] and FASTA [126], and raw data formats such as DICOM‐SB [127]. Standards do not only apply to information. The pipeline in Figure 3 also requires a set of physical standards (in orange), such as SLAS [50], that address the physical objects travelling through the pipeline and their interfacing with the robotic equipment. From an error‐control point of view, they help prevent/reduce equipment malfunction and breakage.

The second set of technologies concerns the standardisation of the instructions sent to the automated platforms and protocol description in general. A subtle benefit of automation is to force protocols to be written precisely, before they are implemented, run on the automated platforms and shared. An automated protocol requires writing an unambiguous sequence of actions, and assigning its most important parameters. When this is done, running the protocols on automated platforms involves little human involvement, which reduces potential errors caused by human variability, and fulfils one of the core promises of automation.

This is, unfortunately, not enough. The problems are apparent during protocol transfer and adaptation (from manual to automated, or from one platform to another), which is rarely a straightforward affair. Liquid handling provides examples of these dangers [65, 128]. Issues occur when parameters associated with protocols developed on different platforms are transported to a different setting—leading to an incomplete or a conflicting set of parameters. Another possible source of errors is poorly designed instrument interfaces. Recent approaches, such as programmatically open platforms like Opentrons [60], or platform‐agnostic languages, such as LabOP [129] and PyLabRobot [130] are exciting and very promising technologies in this regard. They still leave open the possibility of incomplete protocol description however—thus introducing confounding variables in the running of the pipeline. There is therefore a clear need for a community‐wide set of good practices and associated communication strategies regarding protocol description. As previously mentioned, it should contain an element dealing with the influence on some of their parameters on the outcome—and the importance of assigning them adequate values.

The last set of technologies concern orchestration—they appear in Figure 3 (bottom section) as an additional layer (in pale red) labelled ‘Orchestrator’. With complex workflows, several plates may be involved and interact with each other. For instance, sacrificial plates may be made for destructive measurements. There may be branching conditions conditional on the values collected during several measurement steps. Complex plate patterns may be used. Finally, engineering biology projects may use several workflows, while working at large scales. The demands in terms of time and cognitive load that are placed onto the human operators rapidly become overwhelming. An orchestrator will be in charge of dispatching the instructions to the various equipment of the biofoundry and ensure that all operations are conducted in the right order and with the right logic. This is a powerful, and much needed, capacity when combined with LabOp protocols for instance.

An orchestrator is software that can execute workflows, assign tasks to resources, and schedules, runs and monitors the tasks [131]. Popular schedulers include Apache Airflow [132], Azkaban [133], and Oozie [134]. They have become very common in data processing applications [135]. But no one has reported their deployment in biofoundries or in the context of processing biological samples. This state of affairs is not altogether surprising, as orchestrators have been developed for data processing applications, and lack the capacity to directly connect to hardware. This situation is, however, unlikely to continue. An initiative to use orchestrators is being conducted at the London Biofoundry—led by the authors of this paper with results yet to be published. The resources of their biofoundry (equipment, staff etc.) were modelled to expand and adapt the functionalities of the orchestrator, while SILA2 was used for the drivers. It is therefore reasonable to expect workflow orchestrators to become mainstays of biofoundries in the years to come.

## 5 DISCUSSION AND CONCLUSIONS

In this paper, we have presented a review of laboratory automation in engineering biology. Adoption of automation is a direct consequence of a common practical need shared by applications in engineering biology: the need to run operations on biological samples at very large scales. We have made the point that replicability should be made a core principle of the design of the automated pipelines processing biological samples at scale, alongside high throughput. In particular, we have shown that replicability should not be seen as an additional burden for automation efforts, but as an essential requirement for the successful unlocking of the benefits of automation. It is from this viewpoint that suggestions have been made for the design of future automated pipelines with error‐prevention in mind.

In the course of this review, several of the drivers behind the evolution of automation in engineering biology have been listed. It can be safely assumed that some of the drivers behind infrastructure evolution will continue to drive the field in the coming years. They are:

- **Better automation of existing tasks and the automation of new tasks on new types of platforms.** This includes more flexible, programmatically open platforms, the greater adoption of solutions like LabOp, and greater sharing of protocols.
- **Better integration and interfacing of automated tasks to allow for ever more complex workflows.** This includes a greater adoption of data formats, as well as communication protocols, such as SILA2, to link devices. Biofoundries will remain the locations of choice for trialling these efforts.
- **Larger adoption of good practices imported from computing and other engineering fields.** This includes large project management, standardised training material, and a greater emphasis on the communication of errors and their control.

Although improvements in each stream of work will be gradual, their compound effect will lead to an inflection point, where infrastructure will be regarded as code (or error‐correcting code when replicability is added into the mix). When this happens, operators will have a totally abstracted interaction with the infrastructure similar to the interaction with a computer. This will be followed in rapid succession by a search for greater fluidity between operators and the infrastructure they command. It is a safe bet that interfaces based on large language models will appear to help drive biofoundries and make the interaction even more fluid.

It is worth noting, that inasmuch as it is possible with biological samples (some transportation or rebuilding may be needed), workflows could be physically distributed and driven from a remote location. This is essential when testing in different facilities is needed.

In contrast to these exciting developments, progress in two important areas of automation in engineering biology is harder to predict.

The first area relates to throughput. Despite the obvious advancements in productivity, throughput remains limited by the available resources—typically the number of robotic platforms used to process the samples in parallel, and possibly staff number. The time it takes to conduct an operation cannot be reduced beyond what the biology allows. In 2016, Rogers and Church [136] contrasted the already enormous capacity of designing and constructing genetic variants (billions of a day, with multiplexing genome engineering techniques such as MAGE [137]), with existing evaluation capacity (a few thousands of variants a day). This makes the assay phase in the DBTL cycle the critical, limiting phase for all engineering applications—one which will require breakthroughs in high‐throughput analytics and automation [138], for instance further miniaturisation. The second area relates to the learn phase of the DBTL cycle. As is often the case with machine learning, lack of data (itself the consequence of limited throughput, and lack of focus on replicability in historic data) is a bottleneck. An alternative would be to use biology in such a way that it selects the most promising regions of designs [139, 140]. Whether these approaches based on directed evolution can be considered part of engineering biology is open to discussion. Using biology in such a manner can nonetheless, be done in a rational, controllable, and albeit limited, way—for instance, as part of a scoping study to bootstrap DBTL cycles when toxicity is an issue [141]. Currently, efforts to develop an ‘*engineering theory of evolution*’ [142], remain a theoretical exercise aiming to place under a common umbrella a large, disparate, set of techniques with proven successes, but patchy predictive power.

We now wish to address the limited focus of the paper—namely on a certain type of applications (mainly microbial), and on automation in biofoundries. A simple reason for this focus is that such applications have, historically, been the ones of most interest to the engineering biology community, and the most active regarding automation. In their 2023 polemic, Hanson and De Lorenzo [143] warn against a myopic focus on such applications and solutions—highlighting quite rightly the bottleneck in the testing phase, and more controversially limitations of the DBTL approach. This deserves a response.

It is our belief that the principles discussed in this paper, in the context of biofoundries, naturally extend to other settings and applications in engineering biology. First, the idea of biofoundries has not been marketed as being unique. Biofoundries are nonetheless essential components of a wider ecosystem of facilities supporting the bioeconomy [75]. It is therefore not incompatible with dedicated testing facilities. Second, consider another field of application of engineering biology with enormous implications for humanity: agriculture. Agriculture is amenable to automation—driverless, GPS‐tracked tractors are in wide use already. There are also moves to push agriculture in a direction where crops are grown in optimised environments, identified after a few rounds down a DBTL cycle, either in indoor settings or in greenhouses. Finally, agricultural research also has its own type of testing facilities. They are not organised around microbial cultures like biofoundries—but necessitate much larger areas of land, operate very long experiments and are subject to different regulations due to their interaction with the outside environment instead. Rothamsted Research [144] (in Harpenden, UK) is a good example of such facilities. Founded in 1843, it is one of the oldest agricultural research institutions in the world—and has been a major player in the British (pre‐)bioeconomy landscape since then. It is also widely regarded as a shining example of integration in a pre‐computer age. In 1919, the institution hired Ronald Fisher to analyse the vast (for the time) amount of data they collected. This, in turn, led to the creation of a theory that lies at the heart of modern statistics: experimental design—a theory further developed by many of the luminaries who later worked at the institution.

In the end, the fundamentals of the problems remain identical. The physical and time scales may vary, as will the multiple facilities needed for building, testing, learning and ultimately producing. But the method for solving the problems remains the same: iterative searches in a design space, as described with the DBTL cycle. In the absence of reliable modelling, this means building and testing a very large number of samples—and, nowadays, using high‐throughput automation. High throughput and replicability are bound together in a co‐dependent, symbiotic relationship. Practitioners of engineering biology should therefore treat them accordingly—and do so from the very start of their projects.

## AUTHOR CONTRIBUTIONS

**Matthieu Bultelle**: Writing – original draft; Writing – review & editing. **Alexis Casas**: Writing – review & editing. **Richard Kitney**: Supervision; writing – review & editing.

## CONFLICT OF INTEREST STATEMENT

Neither co‐Editor‐in‐Chief was involved in the handling of the article or its peer review process. An Associate Editor has taken full responsibility of the editorial process for the article.

[Correction added on 22 Jul 2024, after first online publication. The conflict of interest section is updated.]

## Data Availability

Data sharing not applicable to this article as no datasets were generated or analysed during the current study.

## References

[1] Voigt, C.A. : Synthetic biology 2020–2030: six commercially‐available products that are changing our world. Nat. Commun. 11(1), 6379 (2020). 10.1038/s41467-020-20122-2 33311504 PMC7733420

[2] OECD : Artificial Intelligence in Science: Challenges, Opportunities and the Future of Research’ (Organisation for Economic Co‐operation and Development (2023)

[3] Flores Bueso, Y. , Tangney, M. : Synthetic biology in the driving seat of the bioeconomy. Trends Biotechnol. 35(5), 373–378 (2017). 10.1016/j.tibtech.2017.02.002 28249675

[4] Safeguarding the Bioeconomy. National Academies Press (2020) 32352690

[5] Cambridge Dictionary | English Dictionary. Translations & Thesaurus, https://dictionary.cambridge.org/. Accessed March 2024

[6] Holland, I. , Davies, J.A. : Automation in the life science research laboratory. Front. Bioeng. Biotechnol. 8 (2020). 10.3389/fbioe.2020.571777 PMC7691657 33282848

[7] Frohm, J. , et al.: Levels of automation in manufacturing. Ergon. ‐ Int. J. Ergon. Hum. Factors 30(3) (2008)

[8] UK Synthetic Biology Roadmap coordination Group , et al.: A Synthetic Biology Roadmap for the UK. TSB Technology Strategy Board (2012)

[9] Clarke, L. : Synthetic biology, engineering biology, market expectation. Eng. Biol. 4(3), 33–36 (2020). Wiley Online Library. 10.1049/enb.2020.0021 Accessed March 2024 36968158 PMC9996698

[10] Engineering Biology Leadership Council ‐ Innovate UK Business Connect. https://iuk.ktn‐uk.org/programme/engineering‐biology‐leadership‐council/, Accessed March 2024

[11] Endy, D. : Foundations for engineering biology. Nature 438(7067), 449–453 (2005). 10.1038/nature04342 16306983

[12] Andrianantoandro, E. , et al.: Synthetic biology: new engineering rules for an emerging discipline. Mol. Syst. Biol. 2(1), 2006.0028 (2006). 10.1038/msb4100073 PMC1681505 16738572

[13] Clarke, L.J. : Synthetic biology UK: progress, paradigms and prospects. Eng. Biol. 1(2), 66–70 (2017). 10.1049/enb.2017.0022

[14] Yadav, V.G. , et al.: The future of metabolic engineering and synthetic biology: towards a systematic practice. Metab. Eng. 14(3), 233–241 (2012). 10.1016/j.ymben.2012.02.001 22629571 PMC3615475

[15] Xu, P. , et al.: Improving metabolic pathway efficiency by statistical model‐based multivariate regulatory metabolic engineering. ACS Synth. Biol. 6(1), 148–158 (2017). 10.1021/acssynbio.6b00187 27490704

[16] Canton, B. , Labno, A. , Endy, D. : Refinement and standardization of synthetic biological parts and devices. Nat. Biotechnol. 26(7), 787–793 (2008). 10.1038/nbt1413 18612302

[17] Bultelle, M. , de Murieta, I.S. , Kitney, R. : Introducing SynBIS — the synthetic biology information system. In: IET/SynbiCITE Engineering Biology Conference’ IET/SynbiCITE Engineering Biology Conference, pp. 1–2 (2016)

[18] Blazeck, J. , et al.: Controlling promoter strength and regulation in Saccharomyces cerevisiae using synthetic hybrid promoters. Biotechnol. Bioeng. 109(11), 2884–2895 (2012). 10.1002/bit.24552 22565375

[19] Salis, H.M. , Mirsky, E.A. , Voigt, C.A. : Automated design of synthetic ribosome binding sites to control protein expression. Nat. Biotechnol. 27(10), 946–950 (2009). 10.1038/nbt.1568 19801975 PMC2782888

[20] Ajikumar, P.K. , et al.: Isoprenoid pathway optimization for taxol precursor overproduction in Escherichia coli. Science 330(6000), 70–74 (2010). 10.1126/science.1191652 20929806 PMC3034138

[21] Hanson, G. , Coller, J. : Codon optimality, bias and usage in translation and mRNA decay. Nat. Rev. Mol. Cell Biol. 19(1), 20–30 (2018). 10.1038/nrm.2017.91 29018283 PMC6594389

[22] Naseri, G. , Koffas, M.A.G. : Application of combinatorial optimization strategies in synthetic biology. Nat. Commun. 11(1), 2446 (2020). 10.1038/s41467-020-16175-y 32415065 PMC7229011

[23] Misawa, N. , et al.: Elucidation of the Erwinia uredovora carotenoid biosynthetic pathway by functional analysis of gene products expressed in Escherichia coli. J. Bacteriol. 172(12), 6704–6712 (1990). 10.1128/jb.172.12.6704-6712.1990 2254247 PMC210783

[24] Myeong, N.R. , et al.: Complete genome sequence of antibiotic and anticancer agent violacein producing *Massilia* sp. strain NR 4‐1. J. Biotechnol. 223, 36–37 (2016). 10.1016/j.jbiotec.2016.02.027 26916415

[25] Spice, A.J. , et al.: Improving the reaction mix of a *Pichia pastoris* cell‐free system using a design of experiments approach to minimise experimental effort. Synth. Syst. Biotechnol. 5(3), 137–144 (2020). 10.1016/j.synbio.2020.06.003 32637667 PMC7320237

[26] Elowitz, M.B. , Leibler, S. : A synthetic oscillatory network of transcriptional regulators. Nature 403(6767), 335–338 (2000). 10.1038/35002125 10659856

[27] Gardner, T.S. , Cantor, C.R. , Collins, J.J. : Construction of a genetic toggle switch in Escherichia coli. Nature 403(6767), 339–342 (2000). 10.1038/35002131 10659857

[28] Group, T.B.F. , et al.: Engineering life: building a FAB for biology. Sci. Am. 294(6), 44–51 (2006). 10.1038/scientificamerican0606-44 16711359

[29] Purnick, P.E.M. , Weiss, R. : The second wave of synthetic biology: from modules to systems. Nat. Rev. Mol. Cell Biol. 10(6), 410–422 (2009). 10.1038/nrm2698 19461664

[30] parts.igem.org. https://parts.igem.org/Main_Page, Accessed March 2024

[31] Kamens, J. : The Addgene repository: an international nonprofit plasmid and data resource. Nucleic Acids Res. 43(D1), D1152–D1157 (2015). 10.1093/nar/gku893 25392412 PMC4384007

[32] McLaughlin, J.A. , et al.: SynBioHub: a standards‐enabled design repository for synthetic biology. ACS Synth. Biol. 7(2), 682–688 (2018). 10.1021/acssynbio.7b00403 29316788

[33] Cardinale, S. , Arkin, A.P. : Contextualizing context for synthetic biology – identifying causes of failure of synthetic biological systems. Biotechnol. J. 7(7), 856–866 (2012). 10.1002/biot.201200085 22649052 PMC3440575

[34] Moschner, C. , Wedd, C. , Bakshi, S. : The context matrix: navigating biological complexity for advanced biodesign. Front. Bioeng. Biotechnol. 10 (2022). 10.3389/fbioe.2022.954707 PMC9445834 36082163

[35] Del Vecchio, D. , et al.: Future systems and control research in synthetic biology. Annu. Rev. Control 45, 5–17 (2018). 10.1016/j.arcontrol.2018.04.007

[36] Chory, E.J. , et al.: Enabling high‐throughput biology with flexible open‐source automation. Mol. Syst. Biol. 17(3), e9942 (2021). 10.15252/msb.20209942 33764680 PMC7993322

[37] Boo, A. , Ellis, T. , Stan, G.‐B. : Host‐aware synthetic biology. Curr. Opin. Syst. Biol. 14, 66–72 (2019). 10.1016/j.coisb.2019.03.001

[38] Yeung, E. , et al.: Biophysical constraints arising from compositional context in synthetic gene networks. Cell Syst 5(1), 11–24.e12 (2017). 10.1016/j.cels.2017.06.001 28734826

[39] Borkowski, O. , et al.: Overloaded and stressed: whole‐cell considerations for bacterial synthetic biology. Curr. Opin. Microbiol. 33, 123–130 (2016). 10.1016/j.mib.2016.07.009 27494248

[40] Nikolados, E.‐M. , et al.: Growth defects and loss‐of‐function in synthetic gene circuits. ACS Synth. Biol. 8(6), 1231–1240 (2019). 10.1021/acssynbio.8b00531 31181895

[41] Zhang, R. , et al.: Topology‐dependent interference of synthetic gene circuit function by growth feedback. Nat. Chem. Biol. 16(6), 695–701 (2020). 10.1038/s41589-020-0509-x 32251409 PMC7246135

[42] Block, D.H.S. , et al.: Regulatory consequences of gene translocation in bacteria. Nucleic Acids Res. 40(18), 8979–8992 (2012). 10.1093/nar/gks694 22833608 PMC3467084

[43] Sauer, C. , et al.: Effect of genome position on heterologous gene expression in Bacillus subtilis: an unbiased analysis. ACS Synth. Biol. 5(9), 942–947 (2016). 10.1021/acssynbio.6b00065 27197833

[44] Ilia, K. , Del Vecchio, D. : Squaring a circle: to what extent are traditional circuit analogies impeding synthetic biology? GEN Biotechnol. 1(2), 150–155 (2022). 10.1089/genbio.2021.0014

[45] Paulsson, J. : Models of stochastic gene expression. Phys. Life Rev. 2(2), 157–175 (2005). 10.1016/j.plrev.2005.03.003

[46] Raj, A. , Oudenaarden, A.van : Nature, nurture, or chance: stochastic gene expression and its consequences. Cell 135(2), 216–226 (2008). 10.1016/j.cell.2008.09.050 18957198 PMC3118044

[47] Bogue, R. : Robots in the laboratory: a review of applications. Ind. Robot Int. J. 39(2), 113–119 (2012). 10.1108/01439911211203382

[48] Pereira, D.A. , Williams, J.A. : Origin and evolution of high throughput screening. Br. J. Pharmacol. 152(1), 53–61 (2007). 10.1038/sj.bjp.0707373 17603542 PMC1978279

[49] Major, J. : Challenges and opportunities in high throughput screening: implications for new technologies. J. Biomol. Screen 3(1), 13–17 (1998). 10.1177/108705719800300102

[50] ANSI/SLAS microplate standards. https://www.slas.org/education/ansi‐slas‐microplate‐standards/ Accessed March 2024

[51] Klumpp, M. , et al.: Readout technologies for highly miniaturized kinase assays applicable to high‐throughput screening in a 1536‐well format. J. Biomol. Screen 11(6), 617–633 (2006). 10.1177/1087057106288444 16760365

[52] Mayr, L.M. , Fuerst, P. : The future of high‐throughput screening. SLAS Discov. 13(6), 443–448 (2008). 10.1177/1087057108319644 18660458

[53] Fox, S. , et al.: High throughput screening 2002: moving toward increased success rates. J. Biomol. Screen 7(4), 313–316 (2002). 10.1089/108705702320351150 12230884

[54] Glökler, J. , Schütze, T. , Konthur, Z. : Automation in the high‐throughput selection of random combinatorial libraries—different approaches for select applications. Molecules 15(4), 2478–2490 (2010). 10.3390/molecules15042478 20428057 PMC6257267

[55] Jessop‐Fabre, M.M. , Sonnenschein, N. : Improving reproducibility in synthetic biology. Front. Bioeng. Biotechnol. 7 (2019). 10.3389/fbioe.2019.00018 PMC6378554 30805337

[56] Holowko, M.B. , et al.: Building a biofoundry. Synth. Biol. 6(1), ysaa026 (2021). 10.1093/synbio/ysaa026 PMC7998708 33817343

[57] Zielinski, D. , et al.: iPipet: sample handling using a tablet. Nat. Methods 11(8), 784–785 (2014). 10.1038/nmeth.3028 25075904

[58] Najmabadi, P. , Goldenberg, A.A. , Emili, A. : Hardware flexibility of laboratory automation systems: analysis and new flexible automation architectures. JALA J. Assoc. Lab. Autom. 11(4), 203–216 (2006). 10.1016/j.jala.2006.05.014 17416299

[59] Linshiz, G. , et al.: PaR‐PaR laboratory automation platform. ACS Synth. Biol. 2(5), 216–222 (2013). 10.1021/sb300075t 23654257

[60] Opentrons | lab automation | lab robots for life scientists. https://opentrons.com/ Accessed March 2024

[61] May, M. : A DIY approach to automating your lab. Nature 569(7757), 587–588 (2019). 10.1038/d41586-019-01590-z 31110319

[62] Kelwick, R. , et al.: Developments in the tools and methodologies of synthetic biology. Front. Bioeng. Biotechnol. 2 (2014). 10.3389/fbioe.2014.00060 PMC4244866 25505788

[63] Carbonell, P. , Radivojevic, T. , García Martín, H. : Opportunities at the intersection of synthetic biology, machine learning, and automation. ACS Synth. Biol. 8(7), 1474–1477 (2019). 10.1021/acssynbio.8b00540 31319671

[64] Opgenorth, P. , et al.: Lessons from two design–build–test–learn cycles of dodecanol production in Escherichia coli aided by machine learning. ACS Synth. Biol. 8(6), 1337–1351 (2019). 10.1021/acssynbio.9b00020 31072100

[65] Goldman, R.P. , et al.: Highly‐automated, high‐throughput replication of yeast‐based logic circuit design assessments. Synth. Biol. 7(1), ysac018 (2022). 10.1093/synbio/ysac018 PMC9583850 36285185

[66] Edinburgh Genome Foundry. https://onehealthgenomics.ed.ac.uk/facilities‐and‐technologies/edinburgh‐genome‐foundry Accessed March 2024

[67] Hillson, N. , et al.: Building a global alliance of biofoundries. Nat. Commun. 10(1), 2040 (2019). 10.1038/s41467-019-10079-2 31068573 PMC6506534

[68] Si, T. , et al.: Automated multiplex genome‐scale engineering in yeast. Nat. Commun. 8(1), 15187 (2017). 10.1038/ncomms15187 28469255 PMC5418614

[69] Lesaffre: global key player in fermentation. https://www.lesaffre.com/ Accessed March 2024

[70] The organism company. https://www.ginkgobioworks.com/ Accessed March 2024

[71] Tellechea‐Luzardo, J. , et al.: Fast biofoundries: coping with the challenges of biomanufacturing. Trends Biotechnol. 40(7), 831–842 (2022). 10.1016/j.tibtech.2021.12.006 35012773

[72] Watkins, A. , et al.: Public biofoundries as innovation intermediaries: the integration of translation, sustainability, and responsibility. J. Technol. Transf. (2023). 10.1007/s10961-023-10039-5 PMC11341651 39183938

[73] Clarke, L.J. , Kitney, R.I. : Synthetic biology in the UK – an outline of plans and progress. Synth. Syst. Biotechnol. 1(4), 243–257 (2016). 10.1016/j.synbio.2016.09.003 29062950 PMC5625736

[74] Kitney, R. , Freemont, P. : Synthetic biology – the state of play. FEBS Lett. 586(15), 2029–2036 (2012). 10.1016/j.febslet.2012.06.002 22704968

[75] Kitney, R. , et al.: Enabling the advanced bioeconomy through public policy supporting biofoundries and engineering biology. Trends Biotechnol. 37(9), 917–920 (2019). 10.1016/j.tibtech.2019.03.017 31036350

[76] Farzaneh, T. , Freemont, P.S. : Biofoundries are a nucleating hub for industrial translation. Synth. Biol. 6(1), ysab013 (2021). 10.1093/synbio/ysab013 PMC8546609 34712838

[77] SynbiCITE. http://synbicite.com/ Accessed March 2024

[78] Otero‐Muras, I. , Carbonell, P. : Automated engineering of synthetic metabolic pathways for efficient biomanufacturing. Metab. Eng. 63, 61–80 (2021). 10.1016/j.ymben.2020.11.012 33316374

[79] Fernández‐Castané, A. , et al.: Computer‐aided design for metabolic engineering. J. Biotechnol. 192, 302–313 (2014). 10.1016/j.jbiotec.2014.03.029 24704607

[80] Hérisson, J. , et al.: The automated Galaxy‐SynBioCAD pipeline for synthetic biology design and engineering. Nat. Commun. 13(1), 5082 (2022). 10.1038/s41467-022-32661-x 36038542 PMC9424320

[81] Smucker, B. , Krzywinski, M. , Altman, N. : Optimal experimental design. Nat. Methods 15(8), 559–560 (2018). 10.1038/s41592-018-0083-2 30065369

[82] Gilman, J. , et al.: Statistical design of experiments for synthetic biology. ACS Synth. Biol. 10(1), 1–18 (2021). 10.1021/acssynbio.0c00385 33406821

[83] Sieow, B.F.‐L. , et al.: Synthetic biology meets machine learning. In: Selvarajoo, K. (ed.) Computational Biology and Machine Learning for Metabolic Engineering and Synthetic Biology, pp. 21–39. Springer US (2023) 10.1007/978-1-0716-2617-7_2 36227537

[84] SiLA rapid integration. https://sila‐standard.com/ Accessed March 2024

[85] SiLA2 | GitLab. https://gitlab.com/SiLA2 Accessed March 2024

[86] Porr, M. , et al.: Implementing a digital infrastructure for the lab using a central laboratory server and the SiLA2 communication standard. Eng. Life Sci. 21(3–4), 208–219 (2021). 10.1002/elsc.202000053 33716619 PMC7923558

[87] Yeoh, J.W. , et al.: SynBiopython: an open‐source software library for Synthetic Biology. Synth. Biol. 6(1), ysab001 (2021). 10.1093/synbio/ysab001

[88] Synthace: the life science experiment platform for R&D teams. https://www.synthace.com Accessed March 2024

[89] Vrana, J. , et al.: Aquarium: open‐source laboratory software for design, execution and data management. Synth. Biol. 6(1), ysab006 (2021). 10.1093/synbio/ysab006 PMC8209617 34151028

[90] Storch, M. , Haines, M.C. , Baldwin, G.S. : DNA‐BOT: a low‐cost, automated DNA assembly platform for synthetic biology. Synth. Biol. 5(1), ysaa010 (2020). 10.1093/synbio/ysaa010 PMC7476404 32995552

[91] Walsh, D.I. , et al.: Standardizing automated DNA assembly: best practices, metrics, and protocols using robots. SLAS Technol. Transl. Life Sci. Innov. 24(3), 282–290 (2019). 10.1177/2472630318825335 PMC6819997 30768372

[92] Ma, Y. , et al.: Automated high‐throughput DNA synthesis and assembly. Heliyon 10(6), e26967 (2024). 10.1016/j.heliyon.2024.e26967 38500977 PMC10945133

[93] Bryant, J.A., Jr. , et al.: AssemblyTron: flexible automation of DNA assembly with Opentrons OT‐2 lab robots. Synth. Biol 8(1), ysac032 (2023). 10.1093/synbio/ysac032 PMC9832943 36644757

[94] National Academies of Sciences, Engineering, and Medicine; Policy and Global Affairs; Committee on Science, Engineering, Medicine, and Public Policy; Board on Research Data and Information; Division on Engineering and Physical Sciences; Committee on Applied and Theoretical Statistics; Board on Mathematical Sciences and Analytics; Division on Earth and Life Studies; Nuclear and Radiation Studies Board; Division of Behavioral and Social Sciences and Education; Committee on National Statistics; Board on Behavioral, Cognitive, and Sensory Sciences; Committee on Reproducibility and Replicability in Science : Reproducibility and Replicability in Science. National Academies Press (US) (2019)

[95] Ioannidis, J.P.A. : Why most published research findings are false. PLoS Med. 2(8), 4–13 (2005). 10.1080/09332480.2019.1579573 PMC1182327 16060722

[96] Baker, M. : 1,500 scientists lift the lid on reproducibility. Nature 533(7604), 452–454 (2016). 10.1038/533452a 27225100

[97] How science goes wrong. Economist (2013)

[98] McNutt, M. : Reproducibility. Science 343(6168), 229 (2014). 10.1126/science.1250475 24436391

[99] Plesser, H.E. : Reproducibility vs. Replicability: a brief history of a confused terminology. Front. Neuroinf. 11, 76 (2018). 10.3389/fninf.2017.00076 PMC5778115 29403370

[100] Artifact Review and Badging ‐ Current. https://www.acm.org/publications/policies/artifact‐review‐and‐badging‐current Accessed March 2024

[101] Lux, M.W. , Strychalski, E.A. , Vora, G.J. : Advancing reproducibility can ease the “hard truths” of synthetic biology. Synth. Biol. 8(1), ysad014 (2023). 10.1093/synbio/ysad014 PMC10640854 38022744

[102] Beal, J. , et al.: Quantification of bacterial fluorescence using independent calibrants. PLoS One 13(6), e0199432 (2018). 10.1371/journal.pone.0199432 29928012 PMC6013168

[103] Cole, S.D. , et al.: Quantification of interlaboratory cell‐free protein synthesis variability. ACS Synth. Biol. 8(9), 2080–2091 (2019). 10.1021/acssynbio.9b00178 31386355

[104] Beal, J. , et al.: Robust estimation of bacterial cell count from optical density. Commun. Biol. 3(1), 1–29 (2020). 10.1038/s42003-020-01127-5 32943734 PMC7499192

[105] Liu, Y. , Beyer, A. , Aebersold, R. : On the dependency of cellular protein levels on mRNA abundance. Cell 165(3), 535–550 (2016). 10.1016/j.cell.2016.03.014 27104977

[106] Bown, C.P. , Bollyky, T.J. : How COVID‐19 vaccine supply chains emerged in the midst of a pandemic. World Econ. 45(2), 468–522 (2022). 10.1111/twec.13183 34548749 PMC8447169

[107] Louden, E.M. : Scaling up the global COVID‐19 vaccination program: production, allocation, and distribution with an emphasis on equity. Yale J. Biol. Med. 95(3), 379–387 (2022) 36187418 PMC9511941

[108] Macarron, R. , et al.: Impact of high‐throughput screening in biomedical research. Nat. Rev. Drug Discov. 10(3), 188–195 (2011). 10.1038/nrd3368 21358738

[109] Yeow, J.A. , et al.: Effects of stress, repetition, fatigue and work environment on human error in manufacturing industries. J. Appl. Sci. 14(24), 3464–3471 (2014). 10.3923/jas.2014.3464.3471

[110] Anderson, D.I. , et al.: Individual differences in motor skill learning: past, present and future. Hum. Mov. Sci. 78, 102818 (2021). 10.1016/j.humov.2021.102818 34049152

[111] Miller, J.S. , et al.: Micropipetting: an important laboratory skill for molecular biology. Am. Biol. Teach. 66(4), 291–296 (2004). 10.1662/0002-7685(2004)066[0291:mailsf]2.0.co;2

[112] Mante, J. , et al.: Synthetic biology knowledge system. ACS Synth. Biol. 10(9), 2276–2285 (2021). 10.1021/acssynbio.1c00188 34387462

[113] Casas, A. , et al.: Removing the bottleneck: introducing cMatch ‐ a lightweight tool for construct‐matching in synthetic biology. Front. Bioeng. Biotechnol. 9 (2022). 10.3389/fbioe.2021.785131 PMC8784771 35083201

[114] Frohm, J. , Lindström, V. , Bellgran, M. : A model for parallel levels of automation within manufacturing. (2005)

[115] Altschul, S.F. , et al.: Basic local alignment search tool. J. Mol. Biol. 215(3), 403–410 (1990). 10.1006/jmbi.1990.9999 2231712

[116] Kapli, P. , Yang, Z. , Telford, M.J. : Phylogenetic tree building in the genomic age. Nat. Rev. Genet. 21(7), 428–444 (2020). 10.1038/s41576-020-0233-0 32424311

[117] Bultelle, M. , Casas, A., Kitney, R. : Development of an automated standard for lycopene ‐ wider lessons for the development of automated protocols ‐ submitted 17‐Jan‐2024 ‐ in review. ACS Synth. Biol. (2024)., Submitted 17‐Jan‐2024‐In Review

[118] Kong, F. , et al.: Automatic liquid handling for life science: a critical review of the current state of the art. J. Lab. Autom. 17(3), 169–185 (2012). 10.1177/2211068211435302 22357568

[119] Ewald, K. , Eppendorf, A. : Influence of Physical Parameters on the Dispensed Volume of Air‐Cushion Pipett. https://www.eppendorf.com/product‐media/doc/en/112929_Userguide/Eppendorf_Liquid‐Handling_Userguide_021_Reference‐family_Research‐family_Research‐pro_Influence‐physical‐parameters‐dispensed‐volume‐air‐cushion‐pipette.pdf (2015). Accessed March 2024

[120] Batista, E. , Filipe, E. , Mickan, B. : Volume calibration of 10 00 μl micropipettes. Inter‐laboratory comparison. Accred Qual. Assur. 13(4), 261–266 (2008). 10.1007/s00769-008-0362-1

[121] Albert, A. : Minimizing Liquid Delivery risk: automated Liquid handlers as sources of error. Am. Lab. 39(13), 8 (2007)

[122] Engineering Biology Facilities. https://www.londonbiofoundry.org Accessed March 2024

[123] Da Silva, B.A. , Krishnamurthy, M. : The alarming reality of medication error: a patient case and review of Pennsylvania and National data. J. Community Hosp. Intern. Med. Perspect. 6(4), 31758 (2016). 10.3402/jchimp.v6.31758 27609720 PMC5016741

[124] Peccoud, J. , et al.: Organizing laboratory information to analyze the reproducibility of experimental workflows. bioRxiv, 2022–04 (2022)

[125] Galdzicki, M. , et al.: The Synthetic Biology Open Language (SBOL) provides a community standard for communicating designs in synthetic biology. Nat. Biotechnol. 32(6), 545–550 (2014). 10.1038/nbt.2891 24911500

[126] Lipman, D.J. , Pearson, W.R. : Rapid and sensitive protein similarity searches. Science 227(4693), 1435–1441 (1985). 10.1126/science.2983426 2983426

[127] Sainz de Murieta, I. , Bultelle, M. , Kitney, R.I. : Toward the first data acquisition standard in synthetic biology. ACS Synth. Biol. 5(8), 817–826 (2016). 10.1021/acssynbio.5b00222 26854090

[128] Chavez, M. , Ho, J. , Tan, C. : Reproducibility of high‐throughput plate‐reader experiments in synthetic biology. ACS Synth. Biol. 6(2), 375–380 (2017). 10.1021/acssynbio.6b00198 27797498

[129] Bartley, B. , et al.: Building an open representation for biological protocols. ACM J. Emerg. Technol. Comput. Syst. 19(3), 1–21 (2023). 10.1145/3604568

[130] Wierenga, R.P. , et al.: PyLabRobot: an open‐source, hardware‐agnostic interface for liquid‐handling robots and accessories. Device 1(4), 100111 (2023). 10.1016/j.device.2023.100111

[131] Garg, S. , Wang, S. , Ranjan, R. : Orchestration Tools for Big Data (2018)

[132] Apache Airflow. https://airflow.apache.org/ Accessed March 2024

[133] Azkaban. https://azkaban.github.io/ Accessed March 2024

[134] Oozie ‐ Apache Oozie Workflow Scheduler for Hadoop. https://oozie.apache.org/ Accessed March 2024

[135] Barika, M. , et al.: Orchestrating big data analysis workflows in the cloud: research challenges, survey, and future directions. ACM Comput. Surv. CSUR 52(5), 1–41 (2019). 10.1145/3332301

[136] Rogers, J.K. , Church, G.M. : Multiplexed engineering in biology. Trends Biotechnol. 34(3), 198–206 (2016). 10.1016/j.tibtech.2015.12.004 26897356

[137] Wang, H.H. , et al.: Programming cells by multiplex genome engineering and accelerated evolution. Nature 460(7257), 894–898 (2009). 10.1038/nature08187 19633652 PMC4590770

[138] Philp, J. : 6 Digitalisation in the bioeconomy: convergence for the bio‐based industries. Digit. Sci. Technol. Innov, 143 (2020)

[139] Cobb, R.E. , Sun, N. , Zhao, H. : Directed evolution as a powerful synthetic biology tool. Methods 60(1), 81–90 (2013). 10.1016/j.ymeth.2012.03.009 22465795 PMC3399045

[140] Wang, Y. , et al.: Directed evolution: methodologies and applications. Chem. Rev. 121(20), 12384–12444 (2021). 10.1021/acs.chemrev.1c00260 34297541

[141] Casas, A. , et al.: PASIV: a pooled approach‐based workflow to overcome toxicity‐induced design of experiments failures and inefficiencies. ACS Synth. Biol. 11(3), 1272–1291 (2022). 10.1021/acssynbio.1c00562 35261238 PMC8938949

[142] Castle, S.D. , Grierson, C.S. , Gorochowski, T.E. : Towards an engineering theory of evolution. Nat. Commun. 12(1), 3326 (2021). 10.1038/s41467-021-23573-3 34099656 PMC8185075

[143] Hanson, A.D. , Lorenzo, V.de : Synthetic biology─high time to deliver? ACS Synth. Biol. 12(6), 1579–1582 (2023). 10.1021/acssynbio.3c00238 37322887 PMC10278163

[144] Rothamsted research: advancing sustainable agriculture. https://www.rothamsted.ac.uk/ Accessed March 2024

